# Cardiovascular outcomes of cancer patients in rural Australia

**DOI:** 10.3389/fcvm.2023.1144240

**Published:** 2023-04-25

**Authors:** Trent D. Williams, Amandeep Kaur, Thomas Warner, Maria Aslam, Vanessa Clark, Rhonda Walker, Doan T. M. Ngo, Aaron L. Sverdlov

**Affiliations:** ^1^ Hunter New England Local Health District, New Lambton, NSW, Australia; ^2^ Hunter Medical Research Institute, Newcastle, NSW, Australia; ^3^ School of Nursing and Midwifery, College of Health Medicine and Wellbeing, University of Newcastle, Callaghan, NSW, Australia; ^4^ Nursing and Midwifery Centre: Hunter New England Local Health District, New Lambton, NSW, Australia; ^5^ Newcastle Centre of Excellence in Cardio-Oncology, New Lambton Heights, NSW, Australia; ^6^ School of Medicine and Public Health, University of Newcastle, Callaghan, NSW, Australia; ^7^ Hunter Medical Research Institute Asthma and Breathing Research Program, Newcastle, NSW, Australia; ^8^ School of Biomedical Sciences and Pharmacy, University of Newcastle, Callaghan, NSW, Australia

**Keywords:** cardio-oncolody, cardiovasccular risk factors, cancer, rural, prevention, acute coronaiy syndrome, heart failiure, atrial fibillation

## Abstract

**Background:**

Cancer and heart disease are the two most common health conditions in the world, associated with high morbidity and mortality, with even worse outcomes in regional areas. Cardiovascular disease is the leading cause of death in cancer survivors. We aimed to evaluate the cardiovascular outcomes of patients receiving cancer treatment (CT) in a regional hospital.

**Methods:**

This was an observational retrospective cohort study in a single rural hospital over a ten-year period (17th February 2010 to 19th March 2019). Outcomes of all patients receiving CT during this period were compared to those who were admitted to the hospital without a cancer diagnosis.

**Results:**

268 patients received CT during the study period. High rates of cardiovascular risk factors: hypertension (52.2%), smoking (54.9%), and dyslipidaemia (38.4%) were observed in the CT group. Patients who had CT were more likely to be readmitted with ACS (5.9% vs. 2.8% *p* = 0.005) and AF (8.2% vs. 4.5% *p* = 0.006) when compared to the general admission cohort. There was a statistically significant difference observed for all cause cardiac readmission, with a higher rate observed in the CT group (17.1% vs. 13.2% *p* = 0.042). Patients undergoing CT had a higher rate of mortality (49.5% vs. 10.2%, *p *≤ 0.001) and shorter time (days) from first admission to death (401.06 vs. 994.91, *p* ≤ 0.001) when compared to the general admission cohort, acknowledging this reduction in survival may be driven at least in part by the cancer itself.

**Conclusion:**

There is an increased incidence of adverse cardiovascular outcomes, including higher readmission rate, higher mortality rate and shorter survival in people undergoing cancer treatment in rural environments. Rural cancer patients demonstrated a high burden of cardiovascular risk factors.

## Background

Cancer and cardiovascular disease (CVD) are the two most common health conditions and the leading causes of death worldwide (1, 2). In Australia, each year the cost of CVD is $5.9 billion and CVD accounts for 11% of all hospitalisations nationally (1). In rural and regional areas of Australia, where 1/3 of Australians reside, cardiovascular outcomes are worse compared with metropolitan patients. Rural patients experience cardiovascular disease at a 20% higher rate compared to metropolitan patients and have worse clinical outcomes (3). Additionally, compared to their metropolitan counterparts, rural patients have a 7% higher mortality - this has remained largely unchanged despite the improvements in cancer treatment (4).

Cancer survivors have up to an 8-fold increased risk of developing CVD (2). Nationally, there are over 400,000 cancer survivors in Australia, and this is expected to increase due to a continuous decline in cancer death rates (3). The progress in cancer diagnosis and treatment resulted in significant improvements in patient outcomes: currently there is a 70% chance of surviving at least 5 years after cancer diagnosis in Australia (3). However, up to 25% of these cancer survivors die from cardiac disease development within 7 years of cancer diagnosis, making it the leading cause of death in cancer survivors (4, 5). This places an increased burden on the health care system resulting in unplanned admissions for the full range of cardiovascular diseases (6–8). There is a paucity of data within Australia examining long term cardiovascular outcomes for patients who have undergone treatment for cancer, in particular for patients who live in rural and regional areas. Given the additional challenges of access to healthcare and speciality care for rural patients, further data may guide improvement in risk stratification and treatment pathways for rural cancer patients.

We aimed to determine if patients undergoing treatment for cancer in a regional centre were readmitted more frequently for CVD, compared to people admitted to hospital but without a cancer diagnosis. Additionally, we aimed to determine if there were differences in mortality outcomes between the two groups. We then aimed to describe the cardiovascular risk factors of the patients in the CT group and the predictors of cardiac readmission. We hypothesized those patients undergoing cancer treatment would have higher rates of adverse cardiovascular outcomes than those patients admitted to hospital without a cancer diagnosis.

## Methods

### Study population and study design

The study was performed using an observational retrospective cohort design in a single rural hospital (Muswellbrook hospital) in Hunter New England Local Health District. All patients admitted to hospital from 17th February 2010 to 19th March 2019 were evaluated: patients receiving CT were compared to all patients admitted to the hospital without a cancer diagnosis. The Hunter New England region of New South Wales, Australia, covers an area of over 131,785 km^2^, a geographical area the size of England, and includes major cities, regional, rural and remote areas. It services a population of approximately one million, of whom approximately 45% live in metropolitan areas and 55% in regional or rural settings, with 5% of the population being Aboriginal and Torres Strait Islander peoples (5). The health district has one level 6 cancer hospital and is supported by 5 regional and rural cancer treatment centres. The cancer treatment centre used in this study is located within a 46-bed rural hospital.

### Data collection

Utilising the electronic cancer treatment records, we identified all patients who had received cancer treatment (administered in an outpatient cancer treatment infusion unit) during this period, cases receiving treatment for non-cancer conditions do not appear in these records. Demographic details, risk factor profile, relevant co-morbid conditions, cancer diagnosis, and treatment commencement date were obtained from the hospital electronic records. The general admission group was determined by identifying consecutive patients admitted to the hospital during the same time period (2010–2019) without a cancer diagnosis. This was done by excluding patients with a relevant cancer ICD-10 (International Statistical Classification of the Diseases and Related Health Problems) code, as a principal diagnosis or one of the first three secondary diagnoses on discharge from hospital. Cancer patients were not routinely screened for optimisation of cardiovascular risk factors. Patients ≥18 years of age were included in both groups.

Outcome data for both groups was sourced from the institutional Cardiac and Stroke Outcomes Unit database, which has previously been described (6) and included hospital readmissions, Emergency department presentation, in-patient death and all-cause mortality - obtained from the state Births Deaths and Marriage Register. In brief, this institutional database records all admissions to all public hospitals within the health district based on ICD codes (6). Readmission with subsequent cardiac diagnosis were included to determine if the administration of CT places an individual at a higher risk of admission for a cardiac cause. Patients' presentations to emergency departments not resulting in admission were excluded. Comorbidities were identified from any ICD-10 code in the first 30 diagnoses on initial oncological evaluation and on discharge documentation of the hospital admission.

We firstly describe the underlying cardiovascular clinical characteristics of the cancer treatment group. We then compared this group with all patients admitted to the hospital without a cancer diagnosis during the corresponding period to determine the impact of cancer therapy has on cardiovascular outcomes. This study was approved by the Hunter New England Health Research Ethics Committee.

#### Statistical methods

Patient characteristics are reported as mean ± SD, median (interquartile range), or number (percentage). Categorical variables are presented as frequencies and percentages of total number of patients. Chi squared analyses were used to examine categorical differences in demographic variables and continuous variables were analysed using analysis of variance. Univariate Logistic (odds ratios) and multivariable logistic regression (adjusted odds ratios) analyses were used to examine the associations for readmission (30 day and 1 year) for: heart failure (HF), acute coronary syndrome (ACS), atrial fibrillation (AF), stroke and all cause cardiac readmissions. Predictor variables were selected based on univariate significance (*p* < 0.1) and clinical relevance. Age and gender were included as covariates in the multivariable logistic regression analyses. The Kaplan–Meier curve was generated for both groups for the outcome of death using days to death. Missing data was considered missing, and cases were excluded listwise. A *p-*value of <0.05 was considered significant**.** Data analysis was conducted using IBM SPSS (version 27, SPSS, Chicago, IL, USA).

## Results

### Clinical characteristics of people undergoing cancer treatment

**Figure 1:**
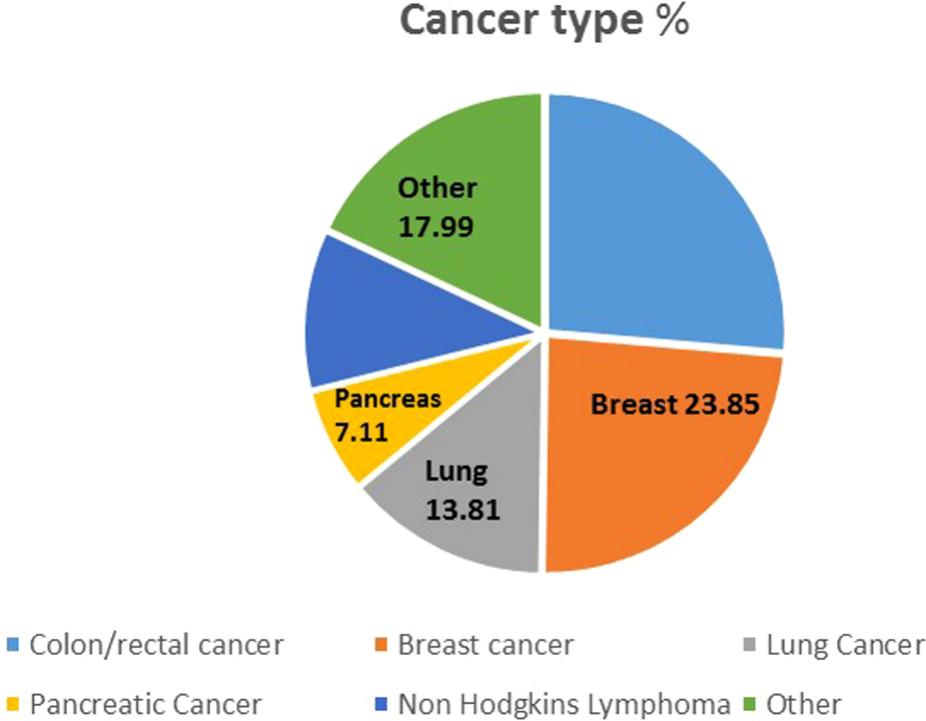
Distribution of cancer types.

A total of 268 patients were administered cancer treatment during the study period whilst 9,304 patients without a current cancer diagnosis were admitted to the hospital during the corresponding period. Colorectal cancers were the major cancers treated, followed by breast and lung cancer (Figure 1).

The cardiovascular co-morbidities, risk factors, demographics and pharmacotherapy of the cancer treatment group are listed in Table 1. This group had a mean age of 63.5 years with 42.5% being male. ACE inhibitors, HMG-CoA reductase inhibitors (statins) and Beta-blockers were the most commonly used cardiovascular pharmacological agents in the CT group. Patients undergoing CT had a high burden of cardiovascular risk factors and co-morbidities. In particular, the modifiable cardiovascular risk factors were common with dyslipidaemia (38.4%), hypertension (52.2%) and prior diabetes (17.5%). Over half of patients reported current or previous tobacco use. In addition, 18.3% of patients had a previous cardiovascular admission. Carboplatin (15.3%), Bevacizumab (13.4%), Capecitabine (9.7%), Trastuzumab (7.5%) were the most frequently used cancer treatment agents, respectively.

**Table 1:** Demographic and cardiovascular characteristics of the chemotherapy lounge patients.

Characteristic *n* (%)	*N* = 268
**Demographics**	
Sex, (Male)	114 (42.5)
Age, mean (SD)	63.5 (13.1)
BSA, mean (SD)	1.9 (0.3)
Current smoker	97 (36.2)
Former smoker	50 (18.7)
Daily alcohol consumption	46 (17.2)
**Medical History**	
Cardiovascular admission	49 (18.3)
PCI	9 (3.4)
CABG	4 (1.5)
Hypertension	140 (52.2)
Diabetes History	47 (17.5)
Dyslipidaemia	103 (38.4)
Ischemic heart disease	18 (6.7)
CVD history	57 (21.3)
Arterial fibrillation	18 (6.7)
Pulmonary hypertension	2 (0.7)
Congestive heart failure	21 (7.8)
COPD/Asthma	37 (13.8)
Chronic Renal Failure	6 (2.2)
Anxiety/Depression	28 (10.4)
Stroke	11 (4.1)
**Cardiovascular Medication History**	
Anti-platelet agents	6 (2.2)
Anticoagulants	15 (5.6)
Beta-blockers	42 (15.7)
ACEIARB	81 (30.2)
HMG-CoA reductase inhibitors	57 (21.3)
Diuretics	19 (7.1)
Calcium channel blockers	8 (3.0)
Anti-Arrhythmic	3 (1.1)
Diabetes Treatment	34 (12.7)

BSA, body surface area; PCI, percutaneous coronary intervention; CABG, coronary artery bypass graft; CVD, cardiovascular disease; COPD, chronic obstructive pulmonary disease; NOAC, non-vitamin K antagonist oral anticoagulants; ACEI, angiotensin-converting enzyme; ARB, angiotensin receptor blockers.

### Readmission and mortality for people undergoing cancer treatment compared to a general admission cohort

**Figure 2:**
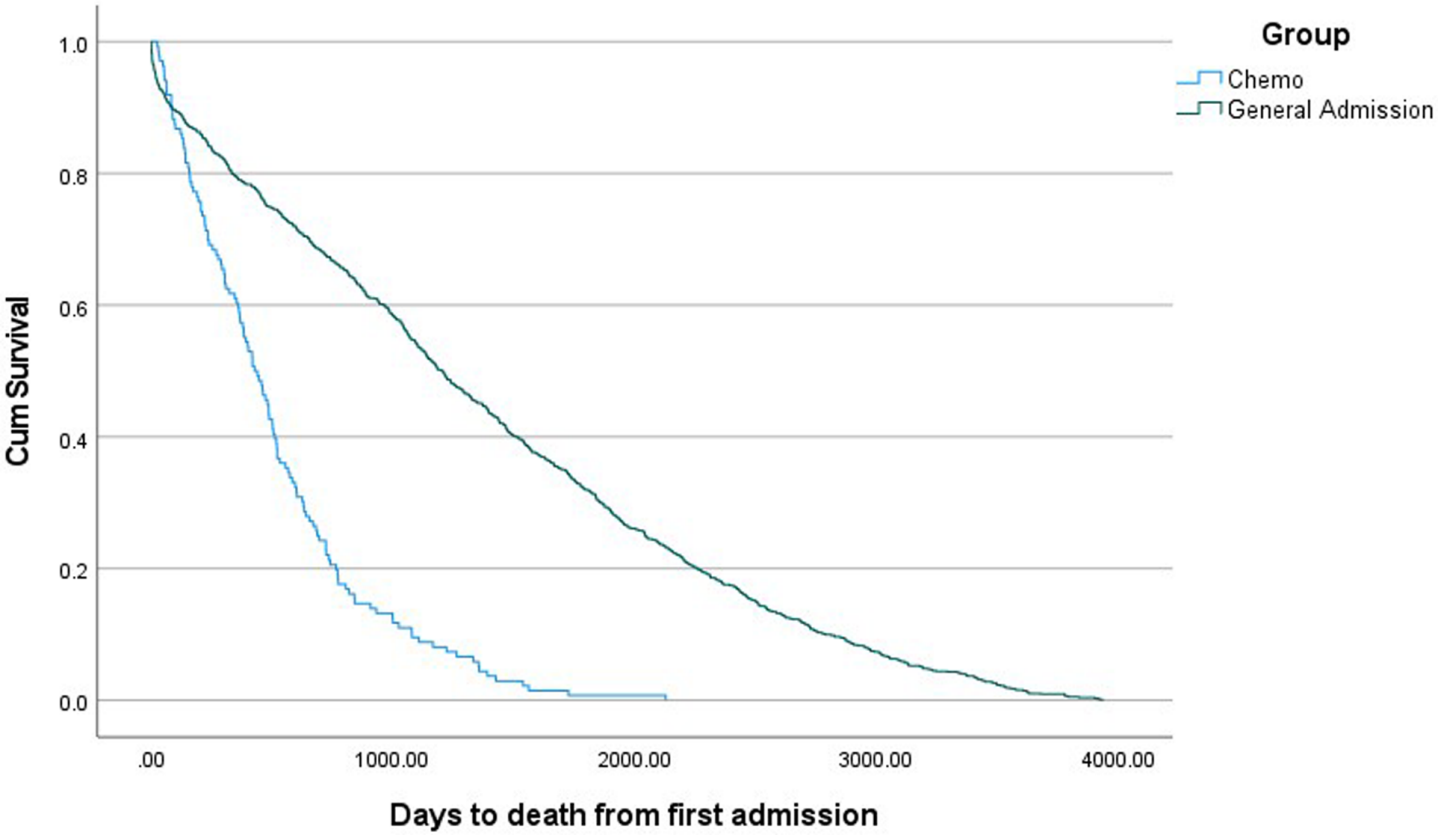
Cumulative survival according to patient group.

Relevant demographic and clinical variables for CT and general admission cohorts are presented in Table 2. Patients in the CT group were older and less likely to be indigenous. No difference in gender and marital status were seen in patients who had CT and general admission cohort. Patients who had CT were more likely to be readmitted within the last year with ACS (5.9% vs. 2.8% *p* = 0.005) and AF (8.2% vs. 4.5% *p* = 0.006) when compared to the general admission cohort. There were also statistically significant differences observed for all cause cardiac readmission, with a higher rate observed in the CT group (17.1% vs. 13.2% *p* = 0.042). There were no differences in 30-day readmission between both groups. Patients undergoing CT had a higher rate of mortality and shorter time from first admission to death (Table 2 and Figure 2).

**Table 2:** Baseline patient characteristics: chemotherapy infusion unit patients vs. general admission patients.

Variable	Group 1 (Chemo *n* = 239)	Group 2 (General admission *n* = 9,304)	*p*
Age (Years), mean, SD	65.40 (12.39)	50.85 (20.452)	**<0** **.** **001**
Gender *n* Male (%)	106 (44.35%)	4,009 (43%)	
Indigenous Status Yes (%)	11 (4.6%)	776 (8.3%)	
**Marital Status *n* (%)**			
Never Married	31 (13.1)	1,924 (20.7)	
Widowed	27 (11.4)	932 (10)	
Divorced	20 (8.4)	472 (5.1)	
Separated	11 (4.6)	303 (3.3)	
Married	147 (62)	5,651 (60.7)	
Not stated	1 (0.4)	21 (0.2)	
**Readmission (30 days)**			
Heart failure – Yes, *N* (%)	0	31 (0.3)	0.412
Acute coronary syndrome Yes, *N* (%)	1 (0.4)	15 (0.2)	0.367
Atrial fibrillation	1 (0.4)	28 (0.3)	0.564
Stroke	0 (0)	7 (0.1)	0.819
All cause cardiac	3 (1.1)	96 (1.0)	0.531
**Readmission (12 months)**			
Heart failure – Yes, *N* (%)	15 (5.6)	459 (4.9)	0.358
Acute coronary syndrome– Yes, *N* (%)	16 (5.9)	259 (2.8)	**0** **.** **005**
Atrial fibrillation	22 (8.2)	414 (4.5)	**0** **.** **006**
Stroke	4 (1.5)	122 (1.3)	0.476
All cause cardiac	46 (17.1)	1,225 (13.2)	**0** **.** **042**
**Mortality Analysis**			
Deceased (Yes), *N* (%)	136 (49.5)	952 (10.2)	**<0** **.** **001**
Days to death from first admission, Mean (SD)	510.13 (401.06)	1,348.36 (994.91)	**<0** **.** **001**

### Predictors of readmission for people undergoing cancer treatment

Those patients who had a prior cardiovascular admission were 3 times more likely to be readmitted with heart failure, however this effect is no longer significant in the multivariable model (Supplementary Table S1). However, prior history of HF remained a significant predictor of future HF admissions even after multivariable adjustment. In addition, the presence of a prior cardiovascular admission, dyslipidaemia, and prior AF and HF placed an individual undergoing CT at higher likelihood of readmission with AF (Supplementary Table S2), however in the multivariable model only prior cardiovascular admission and prior AF remain significant predictors. For all cause cardiac readmission, increasing age, the presence of prior cardiac admission, prior hypertension, prior diabetes, prior dyslipidaemia, prior AF were significant univariate predictors for a subsequent cardiac readmission (Supplementary Table S3). Upon multivariable adjustment, only increasing age and prior AF remained significant predictors of cardiac readmission. Anxiety and stroke were not predictors of readmission after receiving CT (Supplementary Table S3).

Prior use of cardiac medication was examined. Prior ACE/ARB use was associated with higher readmission rate with heart failure, likely a confounder as a surrogate marker of people with high baseline burden of CV disease and risk factors (Supplementary Table S4).

## Discussion

We report on the cardiovascular outcomes of patients receiving cancer treatment at a rural Australian hospital compared to all patients admitted to the same hospital during the study period. This study highlights the potential cardiovascular impacts cancer treatment has on patients on a long-term basis. We noted a high rate of subsequent readmission for AF and ACS in the cancer treatment group. The cancer group demonstrated a higher mortality rate, as well as shorter survival, however we acknowledge this reduction in survival may be driven by the cancer rather than other factors. People receiving CT for advanced cancers are likely to have shorter survival. Furthermore, we noted the high burden of underlying cardiovascular risk factors in those patients undergoing cancer treatment in this rural environment, including smoking history, alcohol consumption, hypertension and high cholesterol. Our study also demonstrated the relatively high rate of prior cardiac hospitalisations in patients having cancer treatment and the higher rate of cardiac readmission for acute coronary syndrome and atrial fibrillation, this is consistent with previous literature and can be explained in part by the common pathophysiology between cancer and cardiovascular disease as well as direct toxic effects of the cancer therapies on the cardiovascular system (7–9).

Our study addresses the two biggest global killers: cancer and cardiovascular disease, focusing on the rural and regional centre and illustrates the health challenges of rural populations. Rural and regional populations have higher cardiovascular risk factor burden (3, 10), and our study is consistent with other studies which demonstrate adverse cardiovascular outcomes in rural and regional populations (11, 12). People who live in rural, regional and remote Australia are likely to have lower life expectancy, with less years of good health, compared to those in major cities (13). In part, this inequality is due to less availability and poorer access to healthcare and health services. Given the noted challenges of timely access to care for both cancer patients and cardiovascular patients, understanding this issue specifically from a regional perspective will assist in informing clinicians and policy makers alike.

Our study demonstrated the high prevalence of cardiovascular modifiable and non-modifiable risk factors in this population. In our study population there was a high incidence of prior cardiovascular admissions in the cancer treatment group, higher than another recently reported large cardio-oncology registry dataset (14). This was additionally reflected in the over representation of modifiable risk factors in our patient group compared to published local risk factor data in cancer patients residing in metropolitan location (5, 15) It is established that cancer and heart disease share common risk factors, however the over representation of cardiovascular risk factors in our group demonstrates a clinical imperative to address this disease burden or implement ongoing surveillance, particularly in under-resourced areas (16). The recent release of the first International Cardio-Oncology treatment guidelines (17) and quality indicators for the prevention and management of cancer therapy-related cardiovascular toxicity (18) represent exciting progress in clinical care. Our real-world analysis further supports the provision of cardio oncology services and the urgent clinical need to implement guideline-based care to prevent or mitigate adverse cardiac complications in all patients living with and beyond cancer.

Many cancer therapies predispose patients to adverse cardiovascular outcomes, including ischaemic heart disease, heart failure, acute coronary syndromes, and atrial fibrillation (19, 20). Furthermore, cancer therapies have also been linked to the development of metabolic syndrome, characterised by changes in glucose tolerance, weight gain and dyslipidaemia, which are in themselves, established risk factors for adverse cardiovascular events (5). Our study demonstrated readmission with a cardiovascular diagnosis, and previous cardiac admission to hospital denoting the presence of concomitant heart disease and risk factors within the cancer treatment population. The lack of guideline-directed use of cardio-protective pharmacological treatment has been previously reported in cancer survivors, despite the fact that this group of patients has high cardiovascular risk (15).

The need for ongoing cardiovascular care in cancer patients is now articulated in international guidelines, previously has been advocated in consensus statements and within the literature (17, 21–23). However, given the challenges of local availability of staff, differing geography, funding arrangements and different health systems, a “one size fits all approach” remains a challenge. These consensus statements and guidelines define the importance of baseline surveillance, care during cancer therapy and in a survivorship capacity. Clearly defined, evidence-based, effective, integrative multi-disciplinary models care exists within cardiovascular disease to assess, monitor and educate patients from diagnosis to long term follow up for a range of other cardiovascular diseases (24–26). Furthermore, while proven cardio-oncology focused models exist, unfortunately these models are not yet widespread internationally (27–29). Additionally, the increasing adoption and prevalence of telehealth models of care represent a potential way to enhance access to timely specialised care for regional, rural and remote communities (30), who are *a priori* at the greatest risk of adverse outcomes. Telehealth approaches are health related services and information that are distributed by electronic and telecommunication technologies. This also allows for long distance clinical care, intervention, monitoring and remote admissions. Multi-disciplinary models of care have shown demonstrable success in rural environments with support from tertiary level hospitals: this may represent a viable model to support the provision of rural cardiology care (31), and be adopted for cardiovascular care for cancer patients. Further studies which assess the models of care to most appropriately provide early risk stratification for cancer patients to prevent the development of and manage pre-existing cardiovascular disease, particularly from a regional context, would provide an important contribution in the care of cancer patients.

## Limitations

Our study has a few limitations: firstly, it is an observational retrospective analysis of prospectively collected data of a relatively small sample size. For the larger dataset of general patients being admitted to hospital, risk factors, cardiotoxicity events, and medications were not collected which may limit comparisons and adjustment of analyses, particularly related to propensity score approach to determine the impact of cardiac risk factors on clinical outcomes. Also, while we have identified cardiovascular risk factors, the degree of their control is unavailable due to limitations in the documentation further highlighting deficiencies in resources and access to appropriate care in regional setting. In addition, due to the granular nature of the data differentiating between patient acuity was not possible. However, this real-world examination of cardiovascular outcomes provides useful data to inform future trials of rural cardiovascular care of cancer patients. We believe our study provides useful real-world data on the adverse cardiovascular outcomes of cancer patients in regional populations, and as such can be applicable in a range of jurisdictions and countries world-wide.

## Conclusion

Our study highlighted the increased incidence of adverse cardiovascular events in people undergoing and following cancer treatment specifically in people living in regional Australia–this has not been previously documented. These data highlight the ongoing need for the provision of cardiovascular focused care in all cancer patients, but especially those with already poorer access to specialised healthcare and prior CVD. Our study suggests the need for consistent recording and management of CVD and CV factors in cancer patients at every stage of the cancer trajectory, from diagnosis into survivorship. The widespread implementation of integrative multi-disciplinary models of care may represent important steps to improve patient care.

## Data Availability

The raw data supporting the conclusions of this article will be made available by the authors, without undue reservation.
