# Supplementary material for: Cardiovascular outcomes of cancer patients in rural Australia
Source: Front Cardiovasc Med. 2023 Apr 25;10:1144240. doi: 10.3389/fcvm.2023.1144240 (PMC10167273; doi:10.3389/fcvm.2023.1144240)
Supplement: Supplementary file 1 [file Datasheet1.docx]

**Supplementary Table 1:** Predictors of heart failure readmission in people undergoing chemotherapy treatment

| **Variable** | **O. R** | **P** | **A.O.R (95% CI)** | **p** |
| --- | --- | --- | --- | --- |
| Age | 1.060 (1.008-1.116) | 0.025 | 1.018 (0.960-1.080) | 0.554 |
| Sex (Male ref) | 1.210 (0.417-3.513) | 0.726 | 1.835 (0.500-6.725) | 0.360 |
| Prior cardiovascular admission | 3.238 (1.094-9.584) | 0.034 | 4.558 (0.559-37.188) | 0.157 |
| Prior hypertension | 1.905 (0.633-5.735) | 0.252 | 1.255 (0.257-6.135) | 0.779 |
| Prior diabetes | 3.433 (1.158-10.182) | 0.026 | 1.541 (0.278-8.541) | 0.621 |
| Prior dyslipidaemia | 1.880 (0.660-5.355) | 0.237 | 0.623(0.142-2.730) | 0.530 |
| Prior atrial fibrillation | 9.750 (2.878-33.033) | <0.001 | 0.228 (0.38-1.354) | 0.104 |
| Prior chronic heart Failure | 20.484 (6.434-65.210) | <0.001 | 0.035 (0.004-0.321) | 0.003 |
| Prior Anxiety/depression | 0.604 (0.076-4.785) | 0.633 | 0.913 (0.097-8.565) | 0.936 |
| Prior stroke | 4.051 (0.793-20.692) | 0.093 | 0.353 (0.042-2.990) | 0.339 |

O.R- Odds Ratio A.O.R- Adjusted Odds ratio (multivariate model)

**Supplementary Table 2**: Predictors of Atrial Fibrillation readmission in people undergoing chemotherapy treatment

| **Variable** | **O. R** | **P** | **A.O.R (95% CI)** | **p** |
| --- | --- | --- | --- | --- |
| Age | 1.075 (1.028-1.126) | 0.002 | 1.058 (0.997-1.123) | 0.061 |
| Sex (Male Ref) | 0.637 (0.264-1.537) | 0.316 | 1.676 (0.532-5.285) | 0.378 |
| Prior cardiovascular admission | 0.105 (0.041-0.272) | <0.001 | 0.170 (0.44-0.656) | 0.010 |
| Prior hypertension | 2.459 (0.923-6.551) | 0.072 | 1.414 (0.369-5.410) | 0.613 |
| Prior diabetes | 2.000 (0.732-5.468) | 0.177 | 1.767 (0.389-8.022) | 0.461 |
| Prior dyslipidaemia | 2.807 (1.120-7.036) | 0.028 | 0.724( 0.210-2.496) | 0.609 |
| Prior atrial fibrillation | 15.795 (5.236-47.648) | <0.001 | 0.132 (0.028-0.629) | 0.011 |
| Prior chronic heart Failure | 6.000 (2.035-17.694) | 0.001 | 1.290 (0.261-6.381) | 0.755 |
| Prior Anxiety/depression | - | 0.998 | - | 0.998 |
| Prior stroke | - | 0.999 | - | 0.999 |

O.R- Odds Ratio A.O.R- Adjusted Odds ratio (multivariate model)

**Supplementary Table 3**: Predictors of All cause cardiac readmission in people undergoing chemotherapy treatment

| **Variable** | **O. R** | **P** | **A.O.R (95% CI)** | **p** |
| --- | --- | --- | --- | --- |
| Age | 1.069 (1.034-1.104) | <0.001 | 1.048 (1.009-1.089) | 0.017 |
| Sex (male) | 0.608 (0.318-1.160) | 0.131 | 0.898 (0.416-1.937) | 0.784 |
| Prior cardiovascular admission | 0.163 (0.080-0.332) | <0.001 | 0.447 (0.162-1.233) | 0.120 |
| Prior hypertension | 2.341 (1.180-4.645) | 0.015 | 1.197 (0.488-2.935) | 0.694 |
| Prior diabetes | 2.597 (1.246-5.412) | 0.011 | 1.033 (0.367-2.911) | 0.951 |
| Prior dyslipidaemia | 2.303 (1.201-4.415) | 0.012 | 0.794 (0.332-1.904) | 0.606 |
| Prior Atrial Fibrillation | 11.324 (3.928-32.640) | <0.001 | 0.262 (0.071-0.971) | 0.045 |
| Prior chronic heart Failure | 13.484 (5.048-36.020) | <0.001 | 0.313 (0.090-1.090) | 0.068 |
| Prior Anxiety/depression | 0.355 (0.81-1.558) | 0.170 | 1.682 (0.356-7.956) | 0.512 |
| Prior stroke | 1.857 (0.473-7.292) | 0.375 | 1.619 (0.311-8.428) | 0.567 |

O.R- Odds Ratio A.O.R- Adjusted Odds ratio (multivariate model)

**Supplementary Table 4**: Pharmacotherapy predictors of Heart failure readmission in people undergoing chemotherapy treatment

| **Variable** | **O. R** | **P** | **A.O.R (95% CI)** | **p** |
| --- | --- | --- | --- | --- |
| Age | 1.060 (1.008-1.116) | 0.025 | 1.045 (0.988-1.107) | 0.126 |
| Sex | 1.210 (0.417-3.513) | 0.726 | 1.3388 (0.436-4.102) | 0.611 |
| Prior Beta-blockers | 2.054 (0.621-6.796) | 0.238 | 1.088 (0.251-4.721) | 0.910 |
| Prior ACE/ARB | 5.029 (1.659-15.238) | 0.004 | 3.936(1.159-13.364) | 0.028 |
| Prior HMG-CoA reductase inhibitors | 1.390 (0.425-4.584) | 0.586 | 0.684 (0.186-2.523) | 0.569 |
| Prior diabetes treatment | 1.067 (0.230-4.955) | 0.934 | 0.602 (0.115-3.163) | 0.549 |

**ACEI** (Angiotensin-converting enzyme)/**ARB** (Angiotensin receptor blockers) O.R- Odds Ratio A.O.R- Adjusted Odds ratio (multivariable model)
